# Maximum Aerobic Function: Clinical Relevance, Physiological Underpinnings, and Practical Application

**DOI:** 10.3389/fphys.2020.00296

**Published:** 2020-04-02

**Authors:** Philip Maffetone, Paul B. Laursen

**Affiliations:** ^1^Independent Researcher, Ormond Beach, FL, United States; ^2^Auckland University of Technology, Auckland, New Zealand

**Keywords:** fatmax, ventilatory threshold, heart rate, health and fitness, exercise training, overfat, fat oxidation, exercise performance monitoring

## Abstract

The earliest humans relied on large quantities of metabolic energy from the oxidation of fatty acids to develop larger brains and bodies, prevent and reduce disease risk, extend longevity, in addition to other benefits. This was enabled through the consumption of a high fat and low-carbohydrate diet (LCD). Increased fat oxidation also supported daily bouts of prolonged, low-intensity, aerobic-based physical activity. Over the past 40-plus years, a clinical program has been developed to help people manage their lifestyles to promote increased fat oxidation as a means to improve various aspects of health and fitness that include reducing excess body fat, preventing disease, and optimizing human performance. This program is referred to as *maximum aerobic function*, and includes the practical application of a personalized exercise heart rate (HR) formula of low-to-moderate intensity associated with maximal fat oxidation (MFO), and without the need for laboratory evaluations. The relationship between exercise training at this HR and associated laboratory measures of MFO, health outcomes and athletic performance must be verified scientifically.

## Introduction

The concept of *maximum aerobic function* began to evolve in the late 1970s as a clinical approach to help a wide range of individuals personalize their health and fitness based on maximizing fat oxidation through exercise, diet and other lifestyle factors (Romain et al., 2012; Randell et al., 2017). The central idea was built upon the premise that early humans oxidized fat as a primary fuel source, associated with a high-quality diet of easily digestible and calorically rich fat and protein with low carbohydrate, necessary, for a larger brain size and extended longevity (Anton et al., 2014; O’Driscoll and Thompson, 2018). In addition, this ability to oxidize large amounts of fat promoted long-term energy requirements for lower-intensity aerobic-based physical activities (Boullosa et al., 2013), and reduced disease risk increasing life expectancy (Finch and Stanford, 2004). Today, similar high fat and protein, and LCD are known to promote very high fat oxidation rates (Volek et al., 2016) with other associated benefits that include weight-loss, reduced cardiometabolic risk, and improved athletic endurance performance (Romain et al., 2012; Maunder et al., 2018c).

Exercise generally increases energy expenditure, but does not necessarily increase fat oxidation, which is influenced by training intensity (Randell et al., 2017). While the rating of perceived exertion and external work completed (distance, power, and velocity) are commonly used to monitor exercise, they do not directly monitor intensity, such as the internal physiological response measured by the HR (Sylta et al., 2014). The optimal intensity for maximal fat oxidation (MFO) varies with an individual’s health, fitness, and other lifestyle stress, particularly the diet (Finch and Stanford, 2004; Romain et al., 2012; Sylta et al., 2014; Volek et al., 2016; Hruby et al., 2017; Maunder et al., 2018c). The development of a personalized exercise HR that was lower in intensity than most other exercise recommendations and promoted MFO can make a positive impact on exercise compliance (Maffetone, 1999), health (Maffetone, 1999, 2010), and athletic performance (Esteve-Lanao et al., 2007; Maffetone and Laursen, 2017). These factors are especially important considering the current global overfat pandemic, defined as excess body fat that impairs health, including downstream cardiometabolic risk factors and chronic diseases (Maffetone and Laursen, 2017, 2020). While many individuals fail to meet minimal exercise guidelines, growing numbers of people who do meet minimal standards for regular aerobic and strength exercise are unsuccessful at reducing excess body fat (Esteve-Lanao et al., 2007; Maffetone and Laursen, 2017, 2020). Overfat conditions have also increased in competitive athletes (Yamamoto et al., 2008; Elliott et al., 2016) and those in active military (Hruby et al., 2017; Gasier et al., 2015).

The human body utilizes a mix of glucose and fatty acids, including ketones, for fuel, which vary considerably from rest to maximal physical efforts, and are highly influenced by exercise intensity, diet, and other lifestyle stress (Finch and Stanford, 2004; Volek et al., 2016; Hruby et al., 2017; Maunder et al., 2018c). The body’s long-term aerobic system relies almost exclusively on fatty acid oxidation as its fuel source, whereas the anaerobic system utilizes both carbohydrate and fat (Hawley and Hopkins, 1995). However, as humans, our dependency on aerobic function and maximizing fat oxidation is a significant aspect of health and fitness (Boullosa et al., 2013).

This article describes the scientific foundation for the maximum aerobic function (MAF) approach, which began in 1977 based on the clinical work of the first author whose range of patients included those who were sedentary, physically active, and amateur age-group and professional athletes (Romain et al., 2012; Randell et al., 2017). Our central aim is to present clinically relevant research findings, practical applications directed at helping individuals increase fat oxidation, and encourage new research on this topic. We used an extensive electronic literature search in the PubMed database for related topics discussed herein, including tracking citations, and our clinical and academic knowledge, professional experience, and previous relevant publications as referenced here.

## Defining Maximum Aerobic Function

The goal of the MAF program has been to help a wide range of individuals personalize their health and fitness, with the central idea of increasing fat oxidation without the need for laboratory testing (Romain et al., 2012; Randell et al., 2017). Individuals using the MAF approach included beginner exercisers, athletes in most sports, those seeking weight-loss, reduced body fat and improved health, and patients undergoing various types of physical rehabilitation (e.g., neuromuscular, cardiac, neurological) (Maffetone, 1999, 2010). Using both clinician-measured and user-reported outcomes, the program emphasized self-care management of lifestyle, including exercise training, diet, and other physical, biochemical and mental-emotional stressors that can significantly influence fat oxidation both at rest and during physical activity (Romain et al., 2012; Sylta et al., 2014; Volek et al., 2016; Randell et al., 2017; Maunder et al., 2018c). Stress responses potentially activate the hypothalamic-pituitary-adrenal (HPA) axis and autonomic nervous system (ANS) sufficiently to increase catabolic and stress hormones, inflammatory cytokines, oxidative stress, and promote other physiological changes that, without proper adaptation, can significantly reduce rates of fat oxidation and impair health and fitness (Siegl et al., 2017).

The emphasis on MFO is important for increasing long-term energy, reducing cardiometabolic risks, and slowing aging (Finch and Stanford, 2004). As the primary site for ATP production, mitochondrial fat oxidation is a significant energy source for skeletal and cardiac muscles, especially during fasting, resting, and low- to moderate-intensity physical activities, and for liver, kidney, adipose, and many other tissues, with ketone bodies, metabolized from fats, being an additional energy source, along with glucose, for the brain (El Bacha et al., 2010; Arts et al., 2015). Increased fat oxidation can also help reduce reactive oxygen species (ROS) production (El Bacha et al., 2010). However, impaired or reduced fat oxidation is a hallmark of aging and disease (Anderson and Weindruch, 2007), and associated with low aerobic capacity, increased fat storage, insulin resistance and other poor health conditions (Heilbronn et al., 2004; Romain et al., 2012; Maunder et al., 2018c).

During the development of the MAF program, various clinical measures were used to monitor outcomes, including blood and urine laboratory testing, and other standard diagnostic measures, such as blood pressure and body fat, along with field tests (described below) (Romain et al., 2012; Randell et al., 2017). A similar clinical outcome by Cao et al. (2019) recently demonstrated positive effects on body fat and waist circumference, HDL cholesterol, blood pressure, maximal oxygen uptake (VO_2_max), and body flexibility following 12 weeks of Fatmax training. In particular, the waist-to-height ratio (WHtR) is an accurate and inexpensive clinical tool for use in the MAF program that is easy to employ, and is a valuable indicator of health and overfat risk for use in all adults and children (Ashwell and Hsieh, 2005), including athletes. The WHtR should be <0.5; the waist should be less than half the height (Esteve-Lanao et al., 2007).

### Determination of the MAF HR

Given the importance of fat oxidation and its relationship to different exercise intensities, an individualized training HR that theoretically was associated with MFO and did not require laboratory assessments was developed (Romain et al., 2012; Randell et al., 2017). Called the *MAF HR*, it resembles the 220-based HR formula (Fox et al., 1971; Emerenziani et al., 2018), and the 6-min walk test (Michael et al., 2017), which estimates HR at VT, but differs from both in its utilization of personal health and fitness information during calculation. In addition, the maximum aerobic function heart rate (MAF HR) is useful as a tool to monitor progress. While this original MAF HR evolved as a clinical tool for patients and athletes as an overall strategy to improve health and fitness (Romain et al., 2012; Randell et al., 2017), over time a formula was developed (discussed later) that resulted in a very similar MAF HR allowing all other individuals to employ it (Fletcher et al., 2017).

The original clinical MAF HR was devised following an extensive evaluation that employed a health and fitness history, physical examinations, and gait analysis at various HR levels during walking or running on an outdoor 400-m track. Pre- and post-outcome measures such as blood and urine tests, body fat measures, as well as training and competitive performance results in athletes were assessed. For example, a group of experienced age-group endurance runners consisting of 223 male and female non-injured adults were instructed to maintain their previous training at or below an assigned personalized MAF HR for 3- to 6-months (Maffetone, 1999; Hoeg and Maffetone, 2015). This was followed by a 5-km road race on a certified course. The results showed that, in addition to developing faster training paces at the same MAF HR, 170 runners improved their race times over previous best performances. These results may be due to increased fat oxidation rates, which can also reduce excess body fat, improve cardiovascular function, and increase VO_2_max (Hetlelid et al., 2015). Increased fat oxidation and decreased submaximal HR (which would increase run speed at the same HR) have been shown respectively by Heatherly et al. (2018), who demonstrated increased fat oxidation rates through a LCD and improved 5 km performance in 6 of 8 participants, and Le Meur et al. (2013) who showed decreased submaximal HR and associated improved competitive performances. In addition, 50% of the variation in Ironman triathlon race time was shown to be explained by peak oxygen uptake and MFO in well trained triathletes competing in the Ironman World Championship (Frandsen et al., 2017).

To implement the general use of the MAF HR, a formula was developed for its determination that did not require a clinical evaluation but was still personalized. Called the *180-Formula*, it is presented in Table 1.

**TABLE 1:** Instructions for determining the MAF HR using the 180-Formula.

1. Subtract your age from 180. 2. Modify this number by choosing one category below that best applies to you: a. If you have or are recovering from a major illness (including any operation or hospital stay), are in rehabilitation, have been prescribed any regular medication, or are chronically overtrained, subtract an additional 10. b. If you are injured, have regressed or not improved in training (such as poor MAF tests) or competition, get more than two colds, flu or other infections per year, have seasonal allergies or asthma, are overfat, are acutely overtraining, or if you have been inconsistent, just beginning or returning to exercise, subtract an additional 5. c. If you have been training consistently (at least four times weekly) for up to 2 years without any of the problems mentioned in (a) or (b), no modification is necessary (use 180 minus age as your MAF HR). d. If you have been training for more than 2 years without any of the problems listed above, have made progress in your MAF tests, and have improved competitively, add 5. The resulting HR is the high end of the HR range with the low being 10 beats below. For example, (a) 40-year old in category (b) would have an exercise range of 125–135 bpm. Users can self-select any intensity within this range.

### Testing the 180-Formula

**FIGURE 1:**
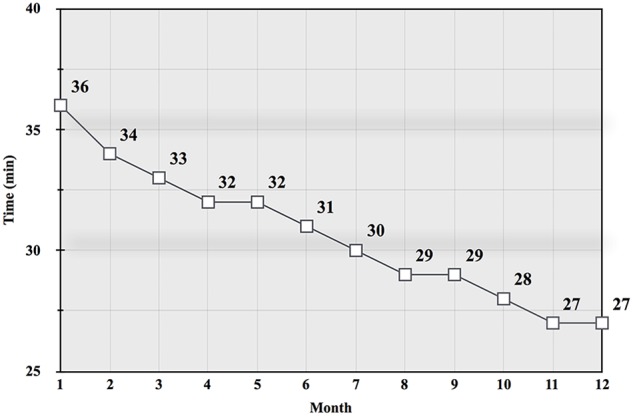
Graph from a 35 year-old experienced male runner’s monthly MAF test results (total time in min, rounded) on a flat 5 km road course during a 12-month aerobic-only (running at or below the MAF HR) training period (Maffetone, 1999).

Monitoring the HR can help avoid increased exercise stress associated with higher intensity (Buchheit, 2014; Siegl et al., 2017) and maintain MFO (Buchheit et al., 2010). Training at the MAF HR over time may also increase work rates at the same HR (Ekblom et al., 1973; Romain et al., 2012; Randell et al., 2017; Fletcher et al., 2017; Purdom et al., 2018). This led to the development of a field test that assessed pace or power at the same MAF HR. Called the *MAF test*, this monthly evaluation can be performed following an easy active warm up, and at the same location, course or using the same equipment (Romain et al., 2012; Randell et al., 2017). Factors that can impact the test include well-known environmental stressors such as altitude and temperature (Bradbury et al., 2019). Figure 1 shows a monthly record of MAF test results, with improvement at the same MAF HR over a 1-year period.

### Comparing the 220 and 180 Formulas

There are important differences between the 220- and 180-Formulas when determining an aerobic training HR. Both formulas begin by considering chronological age: 220 minus age, and 180 minus age. The 220-Formula estimates ones maximum HR (HRmax) from the equation 220 minus age (Fox et al., 1971; Emerenziani et al., 2018). The 180-Formula does not consider HRmax, and 180 minus age is not a meaningful number other than a means to an ending MAF HR. The additional modifications vary with each Formula to arrive at a final training HR range. The 220-Formula uses a range of percentages of 220 minus age, which typically elicits an exercise intensity between 60 and 85% of HRmax. In the 180-Formula, users choose the most appropriate category based on their particular levels of health, fitness, and exercise experience. The 220-Formula’s training HR range could be >40 beats, while the 180-Formula has a 10-heartbeat range. Neither the 220- or 180-Formulas are a replacement for laboratory testing to determine the VT_1_, AerT, Fatmax, or MFO. Table 2 shows two examples comparing each Formula.

**TABLE 2:** The following two examples compare calculations of the 220- and 180-Formulas.

**Example 1: A 35-year old sedentary overfat person beginning an exercise program.**
220-Formula
220−35 × 60−85% = exercise HR range of 111–157
180-Formula
180−35, then
category (b) (−5) = 140; exercise HR range of 130–140
**Example 2: A 35-year old healthy competitive athlete.**
220-Formula
220−35 × 60−85% = exercise HR range of 111–157
180-Formula
180−35, then
category (d) (+ 5) = 150; exercise HR range of 140–150

We can further compare the 220- and 180-Formulas using the data reported in a recent study by Cao et al. (2019). In this study, 30 obese women aged 60–69, who were otherwise healthy and had not exercised for 2 years, had an average exercise HR of 101 ± 9 bpm corresponding to Fatmax. Applying the 220-Formula to this population: 220−64 × 60−85% = the exercise HR range of 94–133. Applying the180-Formula: 180-64−10 = exercise HR range of 96–106.

While the concepts of MAF were first introduced in 1977, similar notions were described earlier. Clinician Kenneth Cooper, who coined the popular exercise term *aerobic*, developed a 12-min running field test in 1968 that showed a good correlation with treadmill-measured maximum oxygen consumption in United States. Air Force male officers (Cooper, 1968). In addition, coach Arthur Lydiard’s *long slow distance* training ideas became popular with many endurance athletes (Lydiard and Gilmour, 2017). However, these concepts did not employ HR monitoring, training was less personalized, other lifestyle factors were usually not considered, and there was no discussion of improving fat oxidation rates. Likewise, the latest recommendations by the United States. Department of Health and Human Services, endorsed by the American College of Sports Medicine, provides guidelines that are general, undefined and not personalized, promoting “moderate-intensity” and “vigorous-intensity” exercise (U.S. Department of Health and Human Services, 2018). In addition, various forms of the 6-min walk test have been used as a biomarker in medicine since the early 1970s, but without always considering factors such as age, sex, height, weight, or other health and fitness conditions, including diet (Heresi and Dweik, 2011). Recently Emerenziani et al. (2019) identified physiological variables in the 6-min walk test that might predict HR at VT in obese men and women, including HR, VO_2_, and individual ventilitory threshold to help determine a Fatmax zone, but did not further individualize health and fitness factors.

While the MAF HR is useful for virtually all those who exercise, it does not replace laboratory testing. However, laboratory testing requires specialized equipment and professional staff, and is unavailable for most individuals, including athletes. This is somewhat remarkable considering the strong association between MFO and improved submaximal and competitive performance (Lucia et al., 1998; Lucia et al., 1999; Aslankeser and Balci, 2017). A combined understanding of the laboratory measures that relate to the concepts of MAF are important and can help promote further research.

## Laboratory Measures of Fat Oxidation

In exercise physiology, various laboratory evaluations of fat oxidation are used to categorize aerobic training status and associated mitochondrial function (Yamamoto et al., 2008). They include MFO, the exercise intensity at which this occurs (Fatmax), the respiratory exchange ratio (RER), and other measures of aerobic metabolism, such the first ventilatory threshold (VT_1_) and/or aerobic threshold (AerT) (Achten et al., 2002; Meyer et al., 2005; Conley et al., 2007; Emerenziani et al., 2019). These evaluations can also help determine an exercise intensity that best corresponds to MFO so that exercise can be prescribed accordingly (Maffetone, 2010; Hruby et al., 2017). However, MFO has been reported to fall across extremely wide ranges, i.e., between 22.6 and 88.8% of VO_2_max (Randell et al., 2017).

Laboratory measures of fat oxidation are usually performed on a treadmill while walking or running, or cycle ergometer, over a stepped range of exercise intensities (Gasier et al., 2015; Hruby et al., 2017). Fatmax is determined as the highest rate of fat oxidation associated with a specific intensity, often represented by the percentage of VO_2_max at which MFO occurs, and is generally associated with MFO at lower versus higher exercise intensities (Achten et al., 2002). This is typically between 45 and 75% of VO_2_max, and can exceed 1.5 g/min (Achten et al., 2002; Volek et al., 2016; Purdom et al., 2018). It is important to note that the transition from low to high exercise causes an increase in non-respiratory CO_2_ excretion (above the lactate threshold), which severely biases gas-exchange estimates of substrate metabolism in favor of carbohydrate over fat oxidation (Rowlands, 2005). This means that fat oxidation reported across most studies making estimates of substrate oxidation rates using gas exchange estimates are likely to be of higher absolute rates (Hetlelid et al., 2015).

In a group of 300 healthy non-athletic men and women, Fatmax was reported to range from 25 and 77% VO_2_max (Venables et al., 2005). In those who are less fit, unhealthy or older, Fatmax can occur at relatively low levels of VO_2_max, sometimes <30% (Jeukendrup and Wallis, 2005; Cao et al., 2019) with the lowest levels in those with chronic disease and in rehabilitation (Meyer et al., 2005; Emerenziani et al., 2016). However, in a study of 1121 athletes participating in various sports, and ranging from 13 to 60 years, Fatmax varied between 22.6 and 88.8% of VO_2_max, with significant positive correlations between MFO (g/min) and Fatmax, and percent body fat (Randell et al., 2017). This further emphasizes the need to individualize exercise prescription to promote increased fat oxidation.

**FIGURE 2:**
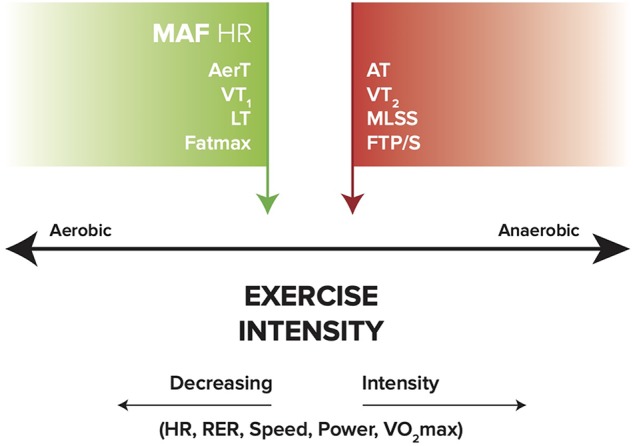
General relationships between aerobic (MAF HR, AerT, VT_1_, LT, and Fatmax), anaerobic (MLSS, FTP/S, VT_2_, and AT), and exercise intensity (HR, RER, speed, power, and VO_2_max). Green arrow indicates MFO; red arrow indicates reduced fat oxidation. MAF HR, maximum aerobic function heart rate; AerT, aerobic threshold; VT_1_, first ventilatory threshold; LT, lactate threshold; MLSS, maximal lactate steady state; FTP/S, functional threshold power/speed; VT_2_, second ventilatory threshold; AT, anaerobic threshold; RER, respiratory exchange ratio; VO_2_max, maximal oxygen uptake.

Laboratory measures of Fatmax, AerT, and VT_1_ appear to generally be associated with MFO and a low to moderate level of exercise intensity (Hawley and Hopkins, 1995; Maffetone, 2010; Sylta et al., 2014; Gasier et al., 2015; Hruby et al., 2017). Theoretically, the MAF HR may be associated with these same measures. In addition, RER decreases with lower intensity and MFO, and increases with reduced fat oxidation at higher intensity levels that approach or exceed anaerobic threshold (AT) (Buchheit et al., 2010; Bradbury et al., 2019). Figure 2 illustrates a simple model of these metrics across the range of exercise intensities.

Exercise can significantly influence laboratory measures of fat oxidation. Training status is positively associated with MFO, with trained individuals generally having greater MFO compared with those who are less trained (Stepto et al., 2001; Nordby et al., 2006; Hetlelid et al., 2015). In a 10 km race performance test, faster runners showed higher fat oxidation rates compared with slower runners (97.4 versus 89.9 g/min, respectively) (Lima-Silva et al., 2010). Lower resting RQ measures may also reflect increased 24-h fat oxidation, and be an important marker for improved health and fitness (Barwell et al., 2009). Exercise success may be indicated by reductions in resting RQ, while increases in RQ in response to weight loss may indicate a predisposition to weight regain after weight loss (Zurlo et al., 1990). In addition to fat oxidation during exercise, 24-h increases in fat oxidation are more important that any one measurement during exercise, including Fatmax, as various non-exercise lifestyle stressors can significantly influence fat oxidation (Buchheit, 2014).

## Influence of Stress on Fat Oxidation

As noted, a variety of physical, biochemical and mental-emotional stressors can, through the actions of the HPA axis and ANS, increase HR, and significantly reduce fat oxidation (Maffetone and Laursen, 2015). While normally observed at higher exercise intensities, excess stress can impair fat oxidation during submaximal exercise too, as indicated by an elevated RER (Febbraio et al., 1998; Watt et al., 2001). Significant reductions in MFO can also occur at very low intensities in individuals with a Fatmax at 22% of VO_2_max (Randell et al., 2017).

**FIGURE 3:**
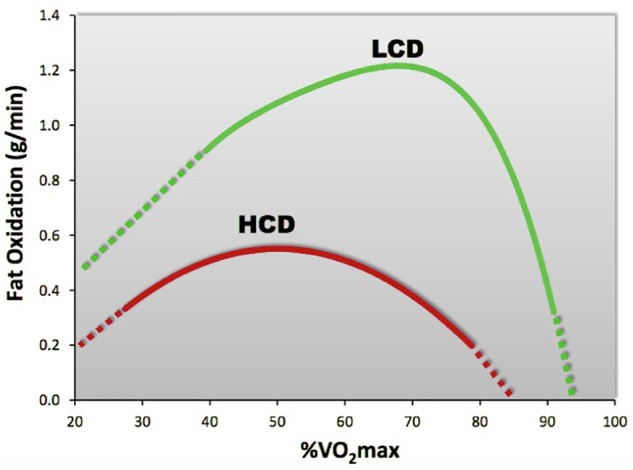
Compared to a high-carbohydrate diet (HCD), a high fat, low-carbohydrate diet (LCD) significantly increases fat oxidation rates. [Adapted from Volek et al. (2016)].

Other stress influences that can cause elevations in exercise HR, potentially reducing MFO, include high environmental temperatures, which raise RER, especially before acclimation (Febbraio et al., 1994), and dehydration (Lambert et al., 1998). A common stress may be the consumption of excess dietary carbohydrates during meals, which can reduce fat oxidation substantially compared with athletes consuming a LCD (Volek et al., 2016). Similarly, the total amount of fat oxidized during exercise is significantly greater before food consumption, as subjects oxidize more fat during exercise performed in the fasted versus post-prandial state (Bennard and Doucet, 2006). While exercise sometimes increases the reward value of food, it can also lead to increased consumption and diminished impact of the exercise effect of body fat loss (Finlayson et al., 2011). Overall, the amounts of dietary carbohydrate and fat intake may make independent contributions to the inter-individual variability of fat oxidation during exercise (Fletcher et al., 2017), with the increased intake of dietary fat and reduced levels of carbohydrates offering maximum benefits (Vogt et al., 2003; Cipryan et al., 2018). In comparison to the more popular high-carbohydrate diet, where Fatmax is typically <0.6 g/min, occurring at ∼50% of VO_2_max, a high-fat, LCD elicits oxidation rates of ∼1.2 g/min, occurring at ∼70% VO_2_max (Volek et al., 2016; see Figure 3).

While excess stress can have other unhealthy consequences such as injuries and overtraining (Siegl et al., 2017), proper adaptation to exercise stress helps promote the well-known health and fitness benefits (Conley et al., 2007). These include reduced sympathetic tone, commonly observed as reductions in resting and exercise HR (Ekblom et al., 1973). However, while moderate doses of training for 3 months may be sufficient to achieve this response, high intensity training may not necessarily lead to greater enhancement of circulatory control nor provide added protective benefit (Iwasaki et al., 2003).

Many exercise prescriptions by coaches and clinicians, like the training routines used by most individuals, employ general guidelines recommended by the American College of Sports Medicine, the World Health Organizations, and other agencies (Garber et al., 2011). These recommendations are usually based on running or walking pace, power output on a bicycle, rowing machine or other equipment, total workout time, or perceived intensity. However, exercising following these metrics can lead to a rising HR (cardiac drift) throughout the workout, the result of increased sympathetic stress (and parasympathetic withdrawal), diminished fat oxidation during and after training, and risk impairing health and fitness, including overtraining (Tank and Lee Wong, 2015). In particular, monitoring cardiovascular exercise stress reactivity is important as this stress is predictive of increased health risks and clinical diseases (Tank and Lee Wong, 2015; Michael et al., 2017).

Increased exercise stress and fatigue leading to cardiac drift can be offset through the biofeedback effect of HR monitoring during exercising (Buchheit, 2014; Siegl et al., 2017). Rather than following general exercise guidelines, or none at all, individuals can monitor the HR to help control cardiac drift and exercise stress to maintain MFO (Buchheit et al., 2010; Romain et al., 2012). This is accomplished at the expense of a gradual slowing of pace or power throughout the workout, with eventual improved physical fitness demonstrated by increased fat oxidation and work rates at the same HR (Romain et al., 2012; Purdom et al., 2018). For example, in professional road cyclists, Lucia et al. (2000) showed increased power outputs at the equivalent VT_1_ HR, alongside improved competitive performances throughout the race season.

Increased stress during higher exercise intensities can also promote excessive lactate and H + accumulation (Febbraio et al., 1998), increasing the risk for peripheral muscle fatigue, reduced excitability and power, central (brain and spinal cord) fatigue, and impaired performance (Amann et al., 2015). Upper or lower body muscle fatigue, across type I and II fibers, can contribute to impaired physical balance (Douris et al., 2011), risking altered gait, reduced performance, and injury. These factors also have the potential to further raise HR, reduce fat oxidation, and contribute to poor exercise responses or affect adherence to a regular routine (Maffetone, 1999, 2010; Rowlands, 2005; Venables et al., 2005).

Social stress is a mental-emotional aspect associated with interactive behavior (Masis-Calvo et al., 2018) and can potentially reduce fat oxidation through the actions of the HPA axis and ANS (Siegl et al., 2017). Social stress can affect individuals exercising in a group, often encouraging competition not unlike herd mentality or collective behavior (Banerjee, 1992; Renfree et al., 2015), which can increase exercise HR (Romain et al., 2012; Randell et al., 2017). Differences may exist between casual exercisers and competitive athletes.

### Casual Exercisers

Compliance and consistency are key features of a successful fitness program, especially in casual and novice exercisers who appear to respond and adhere to their programs better when lower intensities are employed (Douris et al., 2011; Emerenziani et al., 2016). Lower intensity exercise is relatively low risk, individuals are more willing to undertake it, and it is effective, affordable and relatively easy to prescribe (Goodwin, 2010). In addition, *self-selecting* an exercise intensity may also be associated with increased compliance, tends to be lower than or up to VT_1_, with the response to exercise consistently positive, versus the intensity above this threshold where the response to exercise becomes more variable (Ekkekakis, 2003). Self-selection of exercise intensity in men and women also corresponds to that of Fatmax (Dasilva et al., 2011). In addition to subjects with health problems, improved adherence at self-selected lower intensities has also been demonstrated in beginners (Ekkekakis et al., 2005; Aoike et al., 2012).

### Athletes

Unlike casual exercisers, social stress may encourage age-group (amateur) competitive athletes to train at higher intensities, especially with peers (Romain et al., 2012; Randell et al., 2017), due to herd mentality or collective behavior (Banerjee, 1992; Renfree et al., 2015), or adoption of an emotional “no pain, no gain” approach (Maffetone and Laursen, 2019). Higher intensity exercise can increase the risk of overtraining or some of its components (Siegl et al., 2017). For example, age-group athletes may be nearly twice as likely to be affected by stress-related depressive symptoms (Nixdorf et al., 2013). While professional athletes, especially those in endurance sports, are also at risk for overtraining, many have adopted lower intensity training to reduce stress, improve fat oxidation and performance (Sylta et al., 2014). They also have higher levels of MFO and VT_1_, and greater fat oxidation at higher exercise intensities (Lucia et al., 1998; Hetlelid et al., 2015). In addition to improved submaximal performance being associated with successful competition, professional athletes who perform at higher velocities with relatively lower HRs spend significant time at or below VT_1_/Fatmax during long endurance competitive events such as the Hawaiian Ironman or Tour de France (El Bacha et al., 2010; Maunder et al., 2018a, b). During the Tour, for example, Lucia et al. (1999) showed that cyclists spent 70, 23, and 7% at levels <VT_1_, between VT_1_ and second ventilatory threshold (VT_2_), and >VT_2_, respectively, with higher intensities used for the most difficult parts of the race course.

By monitoring exercise HR, all individuals can become more mindful of excessive exercise stress, and help maintain lower intensities that promote MFO and improve health and fitness (Romain et al., 2012; Shaykevich et al., 2015; Randell et al., 2017).

## Future Directions and Conclusion

Exercise that helps promote fat oxidation, reduce excess body fat and improve health and fitness should be simple, easy to implement and adhere to, and be personalized. Accomplishing this on a larger scale without requiring laboratory testing has, to date, been unsuccessful. Despite observed clinical effectiveness of the MAF HR, and its association with MFO, these relationships must be verified scientifically to help address this gap. Encouraging improved health and fitness is a primary public health goal, and the MAF approach can help many individuals better manage their exercise habits to accomplish the objective of reversing the global overfat pandemic, preventing injuries and overtraining, addressing chronic disease, improving quality of life, and reducing rising healthcare costs.

## Data Availability Statement

The raw data supporting the conclusions of this article will be made available by the authors, without undue reservation, to any qualified researcher.

## Author Contributions

PM conceived the theory for the concept of a maximum aerobic function (MAF) and clinical test more than 40 years ago, and wrote the first draft of the manuscript along with a number of edits. PL conceived the need for an academic theory manuscript on MAF that bridged clinical relevance, physiological underpinnings, practical applications, and edited and contributed content to PM’s original work.

## Conflict of Interest

PM is the owner of https://philmaffetone.com/, a company that serves to enhance an individual’s health and exercise performance, and for which the basis of the present theory paper is built upon. PL is co-founder of https://hiitscience.com/, an online education platform that serves to teach the science and application of high-intensity interval training.

The remaining author declares that the research was conducted in the absence of any commercial or financial relationships that could be construed as a potential conflict of interest.
